# Effects of blood glucose level on 18F fluorodeoxyglucose (18F-FDG) uptake for PET/CT in normal organs: an analysis on 5623 patients

**DOI:** 10.1038/s41598-018-20529-4

**Published:** 2018-02-01

**Authors:** Clarice Sprinz, Matheus Zanon, Stephan Altmayer, Guilherme Watte, Klaus Irion, Edson Marchiori, Bruno Hochhegger

**Affiliations:** 1 Department of Nuclear Medicine, Hospital Mãe de Deus - R., Costa, 40, Porto Alegre, Postcode 90110-270 Brazil; 2 Department of Radiology, Pontificia Universidade Católica do Rio Grande do Sul – Av., Ipiranga, 6681, Porto Alegre, Postcode 90619900 Brazil; 3 Medical Imaging Research Lab, LABIMED, Department of Radiology, Pavilhão Pereira Filho Hospital, Irmandade Santa Casa de Misericórdia de Porto Alegre – Av., Independência, 75, Porto Alegre, Postcode 90020160 Brazil; 4 Department of Diagnostic Methods, Federal University of Health Sciences of Porto Alegre - R., Sarmento Leite, 245, Porto Alegre, Postcode 90050-170 Brazil; 5 Department of Radiology, Central Manchester University Hospitals, NHS Foundation Trust - Trust,, Headquarters, Cobbett House, Manchester Royal Infirmary, Oxford Road, Manchester, Postcode M139WL United Kingdom; 6 Department of Radiology, Federal University of Rio de Janeiro Medical School - Av., Carlos Chagas Filho, 373, Rio de Janeiro, Postcode 21941-902 Brazil

## Abstract

Our purpose was to evaluate the effect of glycemia on ^18^F-FDG uptake in normal organs of interest. The influences of other confounding factors, such as body mass index (BMI), diabetes, age, and sex, on the relationships between glycemia and organ-specific standardized uptake values (SUVs) were also investigated. We retrospectively identified 5623 consecutive patients who had undergone clinical PET/CT for oncological indications. Patients were stratified into groups based on glucose levels, measured immediately before ^18^F-FDG injection. Differences in mean SUVmax values among glycemic ranges were clinically significant only when >10% variation was observed. The brain was the only organ that presented a significant inverse relationship between SUVmax and glycemia (p < 0.001), even after controlling for diabetic status. No such difference was observed for the liver or lung. After adjustment for sex, age, and BMI, the association of glycemia with SUVmax was significant for the brain and liver, but not for the lung. In conclusion, the brain was the only organ analyzed showing a clinically significant relationship to glycemia after adjustment for potentially confounding variables. The lung was least affected by the variables in our model, and may serve as an alternative background tissue to the liver.

## Introduction

Positron emission tomography/computed tomography (PET/CT) is an imaging modality used widely for diagnosis, staging, and therapeutic response assessment in oncology^1,2^. Radiolabeled deoxy-2-[18 F]fluoro-D-glucose (^18^F-FDG), a glucose analogue, is the standard tracer used to evaluate neoplastic tissue^3,4^. The standardized uptake value (SUV) is a semiquantitative parameter used to measure tracer accumulation in tissues^2,3^. Although other parameters can be used, SUVmax is the most widely accepted metric given its accuracy and simplicity of application in clinical practice^2,5^.

Several factors, including the administered ^18^F-FDG dosage, length of uptake period, sex and body mass index (BMI), can affect the accuracy of SUV measurement. In addition, clear consensus on the real impact of glycemia on ^18^F-FDG uptake is lacking, as data in the literature regarding the effects of blood glucose on SUVs in different organs are conflicting^5–11^. According to European^12^ and American^13^ guidelines, blood glucose should be measured prior to PET/CT scanning, and examination should be rescheduled whenever the value exceeds 200 mg/dL. However, such rescheduling can be inconvenient for patients and nuclear medicine practices.

SUVs of healthy background tissues, such as the liver and mediastinal blood pool, are commonly used as references to define disease and assess tumor response to therapy^2^. Ideally, these background SUVs should not vary due to glycemic fluctuation at the time of examination, to minimize variability in the assessment of therapeutic response.

The aim of this study was to analyze the effects of blood glucose levels on ^18^F-FDG-uptake in normal organs of interest (brain, liver, and lung). The impacts of other variables, such as sex, age, ^18^F-FDG dose, and BMI, on organ-specific SUVs were also investigated.

## Methods

### Patient population

With the approval of our institutional review board (Institutional Review Board of Hospital Mãe de Deus), we retrospectively identified 5623 consecutive patients from one institution who had undergone clinical PET/CT examinations for oncological indications between March 2010 and March 2013. Written consent was waived by our institutional review board. All procedures performed in studies involving human participants were in accordance with the ethical standards of the institutional research committee and with the 1964 Helsinki declaration and its later amendments. Patients’ medical records were reviewed to collect data on age, sex, weight, diabetes status, use of medications (especially those known to affect glucose metabolism), blood glucose level at the time of ^18^F-FDG injection, injected ^18^F-FDG dose, and time intervals between ^18^F-FDG administration and the initiation of subsequent imaging. Subjects with extensive disease in the target organ and those who had recently taken diabetic medication were excluded from the analysis.

### Preparation for PET/CT

Patients fasted for at least 4 h before imaging and received intravenous ^18^F-FDG injections of 4.62 MBq/kg (0.125 mCi/kg), with a maximum of 555 MBq (15 mCi). Imaging was performed 60–70 min following intravenous ^18^F-FDG injection, as recommended by Wahl *et al*.^2^. Oral contrast was prepared with a mixture of 7.5 mL Omnipaque (Iohexol; Bayer Pharma AG, Berlin, Germany) and 500 mL water before patients were positioned on the PET/CT table.

### Glycemia measurement

Blood glucose levels were measured using a glucometer (Accu-Chek Performa, Accu-Chek Inform II test strips; Roche Diagnostics, Indianapolis, IN, USA) and recorded immediately before ^18^F-FDG injection. Patients were further stratified into four groups based on their serum glucose levels: I) <110 mg/dL, n = 4740; II) 110–159 mg/dL, n = 818; III) 160–179 mg/dL, n = 38; and IV) 180–199 mg/dL, n = 27.

### Image acquisition and reconstruction

All studies were performed with a GEMINI TF PET/CT system (Philips Medical Systems, Cleveland, OH, USA). The Gemini TF is a fully three-dimensional (3D) PET scanner coupled to a 16-slice Brilliance CT scanner. The patient bore is 716 mm in diameter, with an active transverse field of view of 675 mm. The axial field of view per bed position is 180 mm. PET and CT are performed sequentially, with PET acquisition performed in the step-and-shoot mode using an acquisition time of 1.45 s for each bed position. All data were acquired in list mode and reconstructed using a 3D row action maximum likelihood algorithm. The data were corrected for random coincidences, dead time losses, and scatter, and a whole-body low-dose CT scan (120 kVp, 30 mAs/slice) was used for attenuation correction. After positioning, a CT scout image was taken to define the scan range. All patients underwent imaging in the arms-up position from the skull to the upper thighs region. After the completion of CT imaging, the PET scan was performed in the caudal–cranial direction. The scan duration differed according to patient body size, and typically consisted of 12 bed positions. Patients were instructed to breathe shallowly during imaging. The scanner calibration factor is used to convert reconstructed images of patients from scanner units to radioactivity concentration. To reduce variability, the calibration is performed quarterly, and a validation procedure of SUV is performed bimonthly as the manufacturer recommendation.

### Image analysis

The maximum SUV measuements based on body weight (SUVmax) were displayed using the manufacturer’s review workstation (PETview; Philips Medical Systems, Best, The Netherlands). Using the fused transaxial PET/CT images, regions of interest (ROIs) were placed manually on single slices of the right lobe of the liver (diameter, 3 cm), right upper lobe of the lung (diameter, 3 cm), and posterior parietal lobe of the brain (diameter, 1 cm). When these reference regions presented abnormalities, special care was taken to place ROIs in the normal parenchyma. Focal areas of intense FDG uptake, which were considered to represent tumor sites, were not included in these ROIs. For this purpose, CT images were also used. Acquisition of at least one region of interest free of tumor in the organs of interest was possible in all patients.

### Statistical analysis

Data are presented as frequencies and percentages, means ± standard deviations (SDs), or medians and interquartile ranges with minimum and maximum values. We assessed associations between variables with the χ^2^ test. The use of parametric tests was justified by the normal distribution of data, which was evaluated visually and with the Kolmogorov–Smirnov test. For comparison of continuous variables, the ANOVA test was used. We used Dunnett’s test for post-hoc comparisons to assess changes in the three groups with glucose levels ≥110 mg/dL versus the group with glucose <110 mg/dL.

Linear regression models were used to investigate whether demographic components and interventional procedures were associated with ^18^F-FDG uptake in normal organs of interest (brain cortex, liver, and lung). All final multivariate models were adjusted for sex, BMI, activity of ^18^F-FDG injected, and fasting plasma glucose level. Patients’ diabetic status was not included in the model due to correlation with the independent variable glycemia. We considered p values < 0.05 to indicate statistical significance. Analyses were performed using STATA Intercooled 13.1 (STATA Corporation, College Station, TX, USA).

### Effect size and clinical significance interpretation

Effect size was calculated for each group with glucose ≥110 mg/dl by dividing the difference in the group mean SUVmax by the mean SUVmax in the control group using pooled SDs. As the test-retest variability in SUVmax values used with this method has shown to be ~10%^14–16^, differences in mean SUVmax between glycemic ranges were considered to be clinically significant only when >10% variation was observed.

### Data availability statement

The authors declare all data from this study will be provided upon request.

### Ethical approval

All procedures performed in studies involving human participants were in accordance with the ethical standards of the institutional research committee and with the 1964 Helsinki declaration and its later amendments.

## Results

Table 1 depicts the clinical diagnoses of the patients who underwent PET/CT. Lymphoma and sarcomas were the most common diagnoses (32.79%), followed by colorectal, lung and gynecological tumors, which presented similar prevalence (14.19%, 14.00%, and 13.89%, respectively). Table 2 shows demographic information for the study population according to the glycemic ranges. Most patients were not diabetic and had normal glucose values at the time of examination. No difference in gender was present among glycemic categories. However, age, diabetic status, BMI, and activity of ^18^F-FDG injected differed significantly.

**Table 1:** Clinical diagnoses of patients undergoing PET/CT (n = 5623).

Parameter	n	(%)
Lymphoma (HL and NHL) and Sarcomas	1844	(32.79)
Colorectal Cancer	798	(14.19)
Lung Cancer	787	(14.00)
Gynecological Tumors*	781	(13.89)
Melanoma	393	(6.99)
Gastrointestinal tract tumors^†^	202	(3.59)
Genitourinary tract tumors^‡^	197	(3.50)
Head and Neck tumors	169	(3.01)
CNS tumors	79	(1.40)
Without known primary site	73	(1.30)
Other^§^	300	(5.35)

CNS = central nervous system; HL = Hodgkin lymphoma; NHL = non-Hodgkin lymphoma.

^*^Breast, ovarian, uterine, and endometrial.

^†^Pancreas, liver, esophagus, stomach, and biliary tree.

^‡^Kidneys, bladder, ureter, prostate, and testes.

^§^Neuroendocrine, cardiac, thyroid, other.

**Table 2:** Demographic characteristics and PET/CT findings (n = 5623).

Parameter	Total	Cut-off value for fasting plasma glucose (mg/dL)				*P* value
		<110	110–159	160–179	180–199	
Sample size (n)	5623	4740	818	38	27	
Demographic characteristics						
Sex						<0.161
Female	2822 (50.2)	2392 (42.5)	402 (7.1)	20 (0.4)	08 (0.1)	
Male	2801 (49.8)	2348 (41.8)	416 (7.4)	18 (0.3)	19 (0.3)	
Age (years)	56.6 ± 17.3	55.0 ± 17.7	65.4 ± 11.3	66.3 ± 11.0	61.7 ± 17.3	<0.001
Diabetes mellitus						<0.001
No	4858 (86.0)	4619 (82.3)	220 (3.9)	8 (0.1)	3 (0.1)	
Yes	764 (13.5)	112 (2.0).	598 (10.7)	30 (0.5)	24 (0.4)	
BMI (kg/m²)	26.08 ± 4.96	25.70 ± 4.86	28.03 ± 4.93	29.37 ± 5.23	28.55 ± 6.57	<0.001
PET/CT measurements						
Activity injected (mCi)	9.51 ± 2.66	9.42 ± 2.65	9.97 ± 2.73	10.3 ± 1.87	10.1 ± 2.59	<0.001
Cortex SUVmax	10.7 ± 2.90	11.1 ± 2.76	8.53 ± 2.34	5.92 ± 1.86	4.60 ± 1.60	<0.001
Relative difference*		—	(–23.1%)^†^	(–46.7%)^†^	(–58.6%)^†^	
Effect size		—	1.0	2.2	2.9	
Liver SUVmax	2.55 ± 0.36	2.54 ± 0.36	2.64 ± 0.42	2.67 ± 0.42	2.49 ± 0.34	<0.001
Relative difference*		—	( + 3.9%)^†^	( + 5.1%)^†^	(–1.9%)	
Effect size		—	0.25	0.33	0.14	
Lung SUVmax	0.58 ± 0.15	0.58 ± 0.15	0.60 ± 0.14	0.61 ± 0.12	0.61 ± 0.18	<0.002
Relative difference*		—	(3.4%)^†^	(5.2%)	(5.2%)	
Effect size		—	0.14	0.22	0.18	

Data are expressed as mean ± standard deviation or n (%), unless otherwise noted.

BMI = body mass index; PET/CT = positron emission tomography/computed tomography; SUVmax = maximum standardized uptake value.

^*^Calculated as the ratio of the mean SUVmax in each category divided by the mean SUVmax in the glucose <110 mg/dL group.

^†^p < 0.05 in the post-hoc analysis, with glucose <110 mg/dL serving as the control category.

All organs showed significant differences in mean SUVmax among glycemic ranges (p < 0.001; Table 2). The brain cortex was the organ most affected by glycemia, and this effect had a non-linear pattern, as shown by the relative difference in SUVmax between the groups with glucose levels ≥110 mg/dL and that with glucose <110 mg/dL. Post-hoc analysis showed significant differences in cortical SUVmax in all glycemic groups (110–159 mg/dL, −23.1%; 160–179 mg/dL, −46.7%; and 180–199 mg/dL, −58.6%), compared with the control group. In addition, the effect sizes of differences in mean SUVmax were large (>1) for all three groups.

**Figure 1:**
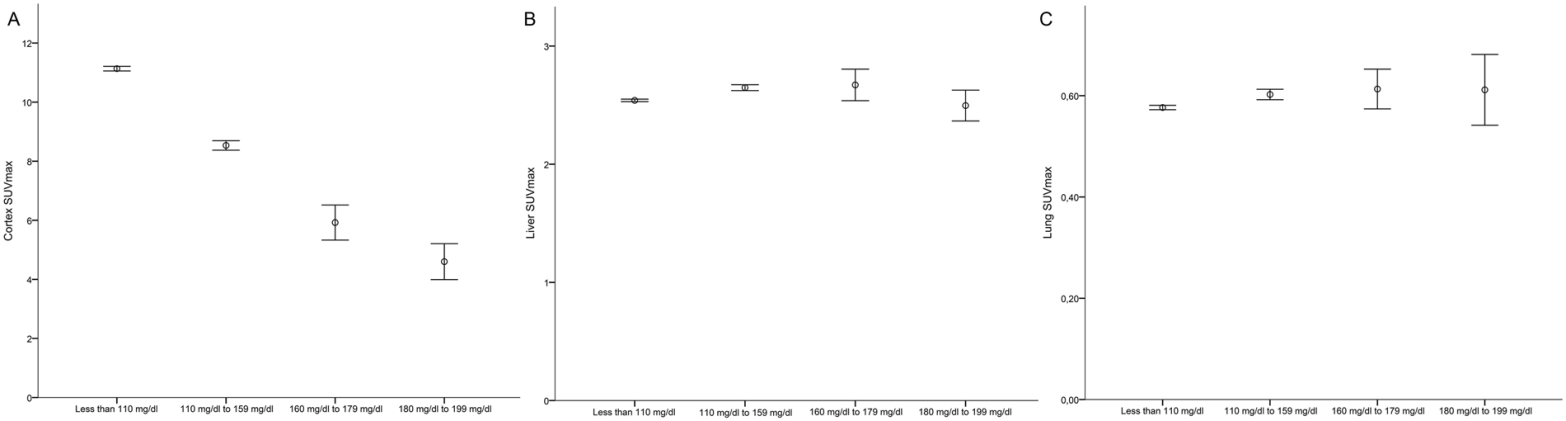
SUVmax values (95% confidence intervals) stratified by glucose range in the cortex, liver, and lung. (**A**) Cortical SUVmax in the population according to glycemic range, showing significant differences among all categories. However, such difference was not observed between SUVs of the liver (**B**) and lung (**C**) and the blood glucose level ranges.

**Figure 2:**
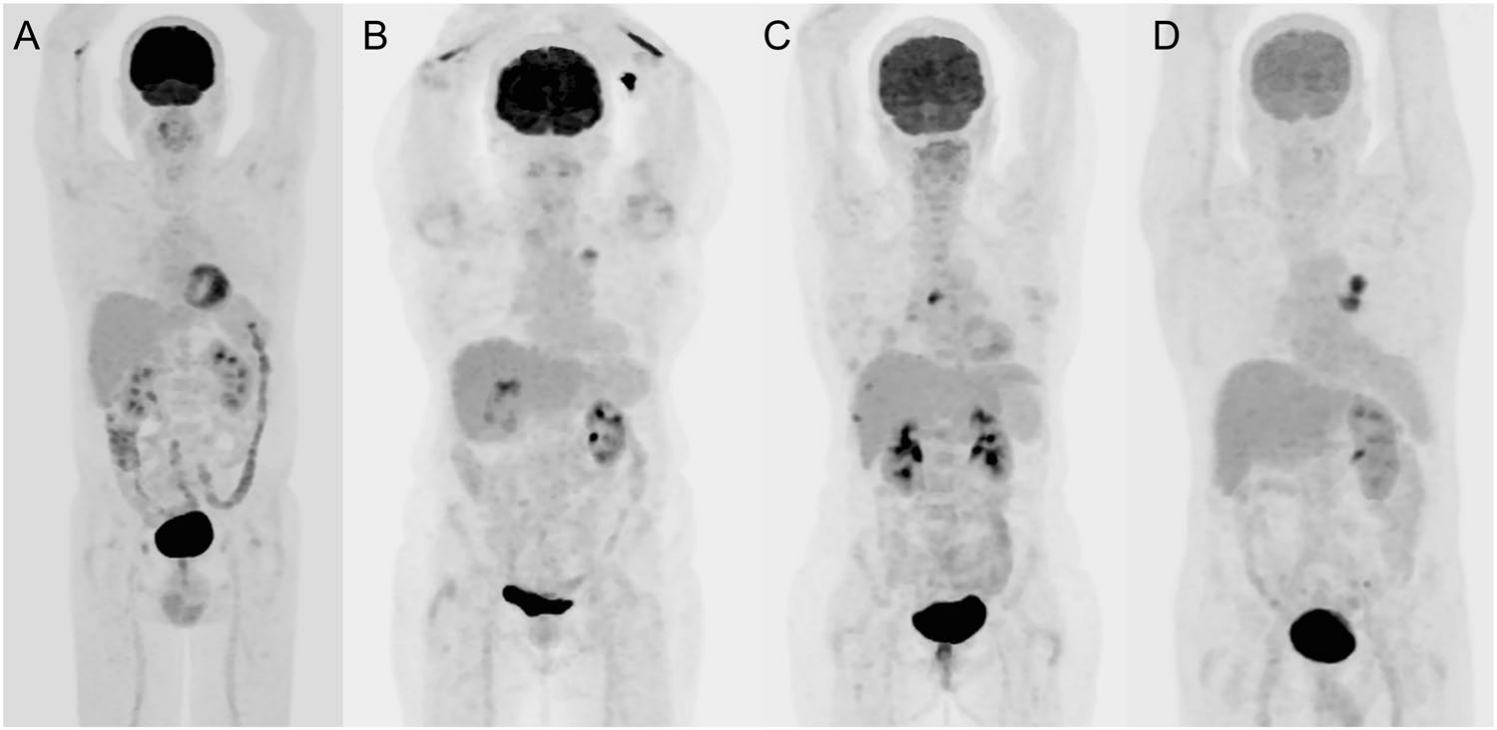
PET images of patients from different glycemia groups: (**A**) <110 mg/dL; (**B**) 110–159 mg/dL; (**C**) 160–179 mg/dL; and (**D**) 180–199 mg/dL. Visual qualitative comparison demonstrates that ^18^F-FDG uptake in the brain was markedly reduced for higher blood glucose levels, whereas no difference is apparent in the liver or lung.

Post-hoc analysis demonstrated significant differences in liver SUVmax for two groups (110–159 and 160–179 mg/dL), and in lung SUVmax for one group (110–159 mg/dL), in comparison with the control group. However, differences in mean SUVmax (<10%) and effect size (≤0.3 and ≤0.22, respectively) were too small to be considered of clinical significance. Figure 1 shows 95% confidence intervals for SUVmax according to blood glucose level ranges in all organs. Visual differences in SUVmax among categories of glycemia are shown in Fig. 2.

In the multivariate analysis adjusted for sex, age and BMI, associations of glycemia with SUVmax remained significant only for the brain cortex and liver, and not for the lung (Table 3). In addition to glycemia, BMI had a significant effect on SUVmax in all organs.

**Table 3:** Results of multivariate regression analysis of glycemia, adjusted for other factors affecting SUV.

Parameter	Cortex		Liver		Lung	
	CE (95% CI)	*P*	CE (95% CI)	*P*	CE (95% CI)	*P*
Male	−0.108 (−0.245, −0.029)	0.105	0.002 (−0.020, 0.017)	0.814	0.000 (−0.007, 0.007)	0.990
BMI (kg/m²)	0.143 (0.127, 0.159)	<0.001	0.026 (0.024, 0.028)	<0.001	0.014 (0.013, 0.015)	<0.001
Activity injected (mCi)	0.002 (−0.027, 0.031)	0.908	0.003 (−0.007, 0.001)	0.155	−0.001 (−0.002, 0.001)	0.536
Glycemia (mg/dL)		<0.001		0.019		0.178
110–159	−2.924 (−3.122, −2.727)		0.049 (0.023, 0.075)		−0.007 (−0.017, 0.004)	
160–179	−5.706 (−6.551, −4.860)		0.046 (−0.064, 0.157)		−0.015 (−0.059, 0.029)	
180–199	−6.961 (−7.949, −5.973)		−0.118 (−0.247, 0.011)		−0.006 (−0.058, 0.047)	

BMI = body mass index; CE = coefficient estimate; CI = confidence interval; SUV = standardized uptake value. All multivariate models were adjusted for sex, body mass index, activity injected, and fasting plasma glucose level.

## Discussion

This study demonstrated that cortex and liver SUVs were affected by glycemia at the time of examination, even after adjusting for sex, age, BMI, and injected dosage of ^18^F-FDG. The effect size of glycemia in the cortex SUVmax was large (>1.0), whereas this effect on ^18^F-FDG uptake in the liver was too small to be clinically significant. On the other hand, the lung was not affected by blood glucose levels in our model. The strengths of this study include the examination of a large sample and the use of a multivariate model to adjust for potentially confounding variables that have rarely been considered in previous studies.

Previous authors have reported a significant impact of glycemia on ^18^F-FDG uptake in the brain cortex. Subjects with higher blood glucose levels presented progressively lower of cortical SUVs^6,17–19^. In our series, cortical ^18^F-FDG uptake showed a significant non-linear relation to glycemia, with average SUVmax differences of up to 58.6% in the higher glycemic ranges compared with the lower ranges, which is consistent with the literature^19^.

Several studies have examined the effect of blood glucose levels on ^18^F-FDG uptake in the liver, with conflicting results reported in the literature. Some authors have reported weak positive correlations between liver uptake and glycemia^7,9,10,19,20^, whereas others have shown no significant association^6,8,21^. In our study, liver SUVmax remained correlated significantly with glycemia, even after adjustment for confounding variables. However, the effect of glycemia on this organ was too small to be of potential clinical relevance (effect size <0.33; relative difference <5.1%).

According to the PET Response Criteria in Solid Tumors (PERCIST), the liver SUV peak corrected for lean body mass (SUL) should be used as a reference for the detection of minimal tumor activity and assessment of response. When the liver is diseased, and thus unsuitable as a background tissue, PERCIST suggest use of the blood-pool activity in the descending aorta as an alternative^2^. However, the latter demands more effort in clinical practice, as multiple ROIs must be defined in the descending aorta. This task could be particularly challenging, especially in diseased vessel walls, such as those affected by atherosclerosis, and in regions of endemic granulomatous disease with compromised mediastinal lymph nodes, as greater metabolic activity due to the inflammatory process could interfere with SUV measurement^22–24^. Although SUVs for other healthy tissues, such as the muscle, heart, lungs, and brain^6,20,23^, have been studied, few authors have proposed organs other than the liver and mediastinum as background tissue for calculation of the tumor-to-background ratio, as an adjunct to monitor the reliability of SUVs.

In our analysis, the lung was the only organ for which correlation of the SUV with glycemia was not significant after adjustment for possible confounding factors, such as sex, BMI, and diabetes status. This finding might be useful, as it suggests that the lung is a stable normal background tissue. On the other hand, in a study by Paquet *et al*., metabolic activity in the lungs was significantly different in PET/CT scans from the same patient done 2.5 to 16 months apart, while metabolic activity remained stable in the liver^23^. However, this retrospective series included a relatively small sample size (n = 70), and further prospective studies with larger samples must test its stability over time to evaluate whether it might serve as an SUV reference for the assessment of tumor treatment response.

Our study has some limitations. Test-retest variability for the assessment of SUV stability in each organ over time was not evaluated. In addition, SUVs corrected for weight, and not SULs, were used.

In conclusion, the brain cortex was the only organ analyzed that showed a clinically significant relationship to glycemia after adjustment for potentially confounding variables. The lung was the organ least affected by the variables in our model, and may serve as an alternative background tissue to the liver.
